# Inhibitors of Glutamate Dehydrogenase Block Sodium-Dependent Glutamate Uptake in Rat Brain Membranes

**DOI:** 10.3389/fendo.2013.00123

**Published:** 2013-09-17

**Authors:** Brendan S. Whitelaw, Michael B. Robinson

**Affiliations:** ^1^Children’s Hospital of Philadelphia Research Institute, Philadelphia, PA, USA; ^2^Departments of Pediatrics and Pharmacology, University of Pennsylvania, Philadelphia, PA, USA

**Keywords:** glutamate, GLT-1, EAAT2, GLAST, GABA, glutamate dehydrogenase, sodium-dependent uptake, epigallocatechin-monogallate

## Abstract

We recently found evidence for anatomic and physical linkages between the astroglial Na^+^-dependent glutamate transporters (GLT-1/EAAT2 and GLAST/EAAT1) and mitochondria. In these same studies, we found that the glutamate dehydrogenase (GDH) inhibitor, epigallocatechin-monogallate (EGCG), inhibits both glutamate oxidation and Na^+^-dependent glutamate uptake in astrocytes. In the present study, we extend this finding by exploring the effects of EGCG on Na^+^-dependent l-[^3^H]-glutamate (Glu) uptake in crude membranes (P2) prepared from rat brain cortex. In this preparation, uptake is almost exclusively mediated by GLT-1. EGCG inhibited l-[^3^H]-Glu uptake in cortical membranes with an IC_50_ value of 230 μM. We also studied the effects of two additional inhibitors of GDH, hexachlorophene (HCP) and bithionol (BTH). Both of these compounds also caused concentration-dependent inhibition of glutamate uptake in cortical membranes. Pre-incubating with HCP for up to 15 min had no greater effect than that observed with no pre-incubation, showing that the effects occur rapidly. HCP decreased the *V*_max_ for glutamate uptake without changing the *K*_m_, consistent with a non-competitive mechanism of action. EGCG, HCP, and BTH also inhibited Na^+^-dependent transport of d-[^3^H]-aspartate (Asp), a non-metabolizable transporter substrate, and [^3^H]-γ-aminobutyric acid (GABA). In contrast to the forebrain, glutamate uptake in crude cerebellar membranes (P2) is likely mediated by GLAST (EAAT1). Therefore, the effects of these compounds were examined in cerebellar membranes. In this region, none of these compounds had any effect on uptake of either l-[^3^H]-Glu or d-[^3^H]-Asp, but they all inhibited [^3^H]-GABA uptake. Together these studies suggest that GDH is preferentially required for glutamate uptake in forebrain as compared to cerebellum, and GDH may be required for GABA uptake as well. They also provide further evidence for a functional linkage between glutamate transport and mitochondria.

## Introduction

Glutamate is the predominant excitatory neurotransmitter in the mammalian CNS and mediates the vast majority of cell-to-cell communication in the brain [for review, see (1)]. In addition to being required for millisecond cell-to-cell communication, plasticity of excitatory synaptic transmission likely underlies learning and memory [for reviews, see (1–3)]. Aberrant glutamatergic transmission has been implicated in a wide variety of neurodevelopmental, neurologic, and psychiatric conditions [for review, see (3)]. For example, an accumulation of glutamate in the extracellular space and the consequent excessive activation of glutamate receptors likely contributes to the cell death that is observed after acute insults to the nervous system, such as stroke and head trauma [for review, see (2)].

Extracellular glutamate is cleared by Na^+^-dependent glutamate transport systems [for reviews, see (4–6)]. In mammals, there are five Na^+^-dependent glutamate transporter gene products; these are called excitatory amino acid transporters (EAAT1-5). EAAT1 (also called GLAST) is found on glia; expression is enriched in cerebellum but also found throughout forebrain (7). EAAT2 (also called GLT-1) is essentially restricted to astroglia with modest expression by a subset of neurons in hippocampus (7, 8). Results from several different types of studies strongly suggest that GLT-1 and GLAST mediate the bulk of glutamate uptake in the mammalian brain [(9); for review, see (10)]. This clearance into astroglia differentiates glutamate from most of the other classical neurotransmitters that are directly recycled back into the presynaptic nerve terminal [for reviews, see (11, 12)]. These transporters co-transport three Na^+^ ions and one H^+^ with glutamate in the inward direction; the cycle is completed with the counter-transport one K^+^ ion (13). With this stoichiometry, these transporters are capable of generating up to a one million-fold concentration gradient of glutamate across the plasma membrane [for review, see (14)].

The astroglial transporters, GLT-1 and GLAST, are enriched on fine astroglial processes near synapses *in vivo* (15, 16). As might be expected, recent studies suggest that these transporters co-compartmentalize with the enzymes/organelles that would be required to efficiently fuel transport in these spatially restricted domains (17). For example, GLT-1 or GLAST co-localize with and physically/functionally interact with the Na^+^/K^+^ ATPase (18). Recently we showed that GLT-1 is part of a co-immunoprecipitable complex with the Na^+^/K^+^-ATPase, most of the glycolytic enzymes, and a subset of mitochondrial proteins (17). We also demonstrated significant co-localization of GLT-1 with a mitochondrial protein *in vivo* and anatomic overlap of mitochondria with GLT-1 in individual astrocytes in organotypic slice cultures. In a subsequent study, we documented similar interactions and anatomic overlap between GLAST and mitochondrial proteins (19). In this later study, we measured the percentage of glutamate that is oxidized in astrocytes. We also examined the effect of an inhibitor of glutamate dehydrogenase (GDH), a mitochondrial enzyme that could contribute to glutamate oxidation, on glutamate uptake and found that it inhibited uptake in astrocytes (19). This effect was not characterized beyond testing of a single concentration of one inhibitor in astrocytes that only express GLAST. In the present study, we characterized the potential effects of inhibitors of GDH on uptake in crude rat brain membranes (P2).

## Materials and Methods

### Materials

Adult male Sprague-Dawley rats were obtained from Charles River (Wilmington, MA, USA). All protocols were reviewed and approved by the Institutional Animal Care and Use Committee of the Children’s Hospital of Philadelphia (Philadelphia, PA, USA). l-[^3^H]Glu (40–80 Ci/mmol), d-[^3^H]Asp (10–25 Ci/mmol), and γ-amino[^3^H]butyric acid ([^3^H]GABA; 70–100 Ci/mmol) were obtained from PerkinElmer (Waltham, MA, USA). The specific activity of all ligands was diluted with non-radioactive l-Glu, d-Asp, or GABA, respectively (Sigma-Aldrich Co., St. Louis, MO, USA). (−)-Epigallocatechin-monogallate (EGCG; ≥95%, from green tea), hexachlorophene (HCP), bithionol (BTH), Hepes, KCl, CaCl_2_, and K_2_HPO_4_ were obtained from Sigma-Aldrich Co. (St. Louis, MO, USA). Tris base, Tris HCl, NaCl, MgCl_2_, dextrose, and sucrose were obtained from Fisher Scientific (Pittsburgh, PA, USA). Tween-20 was obtained from Bio-Rad (Hercules, CA, USA).

### Membrane preparations

Crude membranes (P2) were prepared from cortex and cerebellum as previously described (20). The preparation is commonly referred to as “crude synaptosomal membranes.” In the current paper, we refer to this preparation as crude membranes (P2) to avoid giving the impression that it contains strictly neuronal elements. Cortex or cerebellum was dissected on a metal plate cooled to 4°C. All subsequent steps were performed at 4°C. The tissue was homogenized in 20 volumes (wet weight of tissue) of ice-cold 0.32 M sucrose using a Dounce Teflon/glass homogenizer at 400 rpm for seven strokes (tissue homogenate) and centrifuged at 800 × *g* for 10 min. The supernatant (S1) was then centrifuged at 20,000 × *g* for 20 min. In a subset of experiments, the resultant supernatant was collected (S2). The resultant pellet (P2) was resuspended in 40 vols. of sucrose (0.32 M) by vortexing and centrifuged at 20,000 × *g* for 20 min. This washed crude membrane pellet (P2) was resuspended by vortexing in 50 vols. of sucrose (0.32 M) and homogenized (two strokes at 400 rpm). This resulted in a suspension of approximately 30 μg of protein per 50 μl as determined by the Pierce BCA (bicinchoninic acid) protein assay (Thermo Scientific, Rockford, IL, USA).

### Western blot analyses

The subcellular fractions were mixed with equal volumes of Laemmli sample buffer. Dual color molecular weight marker (Bio-Rad) and 3 or 10 μg of protein from each fraction were resolved on 10% SDS-polyacrylamide gels, and transferred to immobilon FL polyvinylidene fluoride (PVDF) membranes (Millipore, Bedford, MA, USA) as described previously (17, 19). The PVDF membranes were blocked in TBS-T (50 mM Tris, pH 8.0, 150 mM NaCl, 0.1% Tween-20) containing 5% non-fat dry milk for 1 h at 25°C. The membranes were then probed with the appropriate antibody overnight at 4°C: rabbit anti-GLT-1 (1:5,000; Dr. Rothstein), mouse anti-GLAST (1:50; Miltenyi Biotec, Auburn, CA, USA), mouse anti-*N*-methyl-d-aspartate (NMDA) receptor subunit 1 (NR1; 1:500; BD Biosciences, San Jose, CA, USA), rat anti-glial fibrillary acidic protein (GFAP; 1:500; Dr. Lee), or goat anti-neurofilament light polypeptide (NF-L; 1:250; Santa Cruz Biotechnology, Santa Cruz, CA, USA). The membranes were then washed with TBS-T containing 1% milk and incubated with the appropriate fluorescently conjugated anti-mouse, anti-rabbit, anti-goat, or anti-rat antibodies (1:10,000; LiCor Biosciences, Lincoln, NE, USA). Blots were scanned using an Odyssey Infrared Imager (LiCor Biosciences). The yield was calculated as the percentage of total immunoreactivity found in a particular fraction divided by the total immunoreactivity found in the tissue homogenate. The enrichment was calculated as the total immunoreactivity found in a particular fraction divided by the total amount of protein found in the fraction; this was normalized to the tissue homogenate. Therefore a number greater than 1 reflects relative enrichment in a fraction compared to homogenate.

### Transport assays

Sodium-dependent transport of l-[^3^H]Glu, d-[^3^H]Asp, and [^3^H]GABA was measured as previously described (20). Duplicate assays were performed in a final volume of 0.5 ml containing Tris base (5 mM), HEPES (10 mM), NaCl (140 mM), KCl (2.5 mM), CaCl_2_ (1.2 mM), MgCl_2_ (1.2 mM), K_2_HPO_4_ (1.2 mM), dextrose (10 mM), and substrate in the absence or presence of inhibitors (pH = 7.2). In parallel assays, the uptake was measured in the absence of sodium with the substitution of equimolar amounts of choline chloride for NaCl. As HCP and BTH are not readily soluble in aqueous solutions, they were first dissolved in ethanol (HCP) or dimethyl sulfoxide (BTH) as 10 mM stocks. These stocks were diluted such that the same concentration of solvent (0.1% final concentration) was added to each assay; this meant that the highest concentrations of HCP or BTH used in these assays were 10 μM. In all experiments, 0.1% solvent (ethanol or dimethyl sulfoxide) was added to control assays and this concentration had no effect on uptake (data not shown, *n* = 3). EGCG was prepared and diluted in uptake buffer immediately before measuring uptake. All components excluding the crude membranes were combined into 12 mm × 75 mm glass tubes and equilibrated to 37°C. The assay was initiated with the addition of P2 membranes (50 μl) and stopped with 2 ml of ice-cold (4°C) choline-containing buffer after 3 min. For analyses of the effects of pre-incubation with HCP, the assay was initiated with the addition of radioactive substrate to the crude membranes (P2) incubated with HCP. After the addition of cold choline-containing buffer, the assays were filtered onto pre-wetted glass filter paper (FP-100; Brandel, Gaithersburg, MD, USA) using a cell harvester (Brandel, Gaithersburg, MD, USA). Filters were rinsed three times with 2 ml of cold choline-containing buffer. The radioactivity trapped in the membranes was solubilized with 5 ml of Cytoscint ES (MP Biomedicals, Solon, OH, USA) and measured using scintillation spectrometry (Beckman-Coulter Instruments, LS 6500). Sodium-dependent uptake was determined by subtracting the signal in the choline-containing buffer from the signal in the sodium-containing buffer. The total concentration of substrate (radioactive and non-radioactive) was 0.5 μM unless otherwise indicated. The Na^+^-independent signal observed was less than 5% of the total uptake in the presence of sodium.

### Data analysis

All values reported are the mean ± SEM of at least three independent experiments that were performed on different days. Concentration-response curves were fit to one-site and two-site competition curves, and these fits were compared by *F*-test using Prism 5.0 (GraphPad Software Inc., La Jolla, CA, USA). The top of the curves were constrained to 100% (no inhibition), and for all three compounds, the maximal predicted inhibition from the curve fits was essentially 100% (complete inhibition). Kinetic analyses of glutamate transport performed in the absence and presence of HCP were fit by linear regression as Eadie–Hofstee plots. *K*_m_ and *V*_max_ values were compared using one-way ANOVA with a Bonferroni *post hoc* test using InStat (GraphPad Software Inc., La Jolla, CA, USA). The data for l-[^3^H]Glu, d-[^3^H]Asp, and [^3^H]GABA uptake in cortex and cerebellum was analyzed using one-way ANOVA with a Bonferroni *post hoc* test using InStat.

## Results

**Figure 1:**
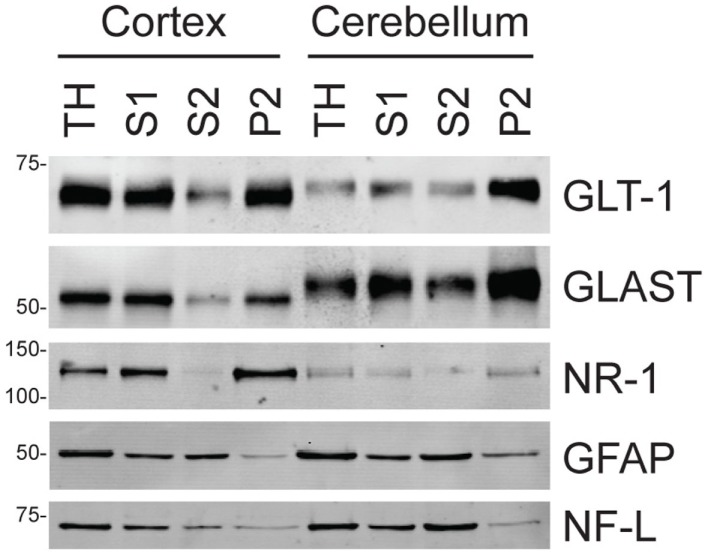
**Analyses of neuronal and astroglial proteins in tissue homogenate (TH) and various subcellular fractions as described in Section “Materials and Methods**.” To ensure that signal was in the linear range, 3 μg of protein from each fraction were used for the analyses of GLT-1 or GLAST and 10 μg of protein were for the analyses of the other proteins (NR1 subunit of the NMDA receptor, NR1; GFAP, and NF-L). The western blots shown are all from one animal; these analyses have been reproduced in three additional animals (see Table 1 for data analyses).

We recently found that EGCG, a compound extracted from green tea that inhibits GDH (21), blocks Na^+^-dependent glutamate uptake in astrocytes. EGCG was only tested at a single concentration (1 mM) using a single concentration of glutamate (19). The goal of the present study was to determine if inhibition of GDH might inhibit Na^+^-dependent glutamate transport in membrane preparations from brain. As astrocytes in culture express GLAST and not GLT-1 (22, 23), we used crude membranes (P2) from cortex to further explore this effect. Genetic deletion of GLT-1 from mice essentially eliminates uptake from crude cortical membrane preparations (P2), and the pharmacology of glutamate uptake in this preparation parallels that observed for GLT-1 (9, 20, 24). Although this is classically considered a subcellular fraction that contains nerve terminals, it also contains substantial amounts of astroglial elements (25, 26). We analyzed the subcellular distributions of two different glial glutamate transporters [GLT-1 and GLAST; (15, 16)], a neuronal receptor [the NR1 subunit of the NMDA receptor; (1)], a glial cytoskeletal protein (GFAP), and a neuronal cytoskeletal protein [NF-L; (27); see Figure 1; Table 1]. The yield of both glutamate transporters was about 40% in the cortical P2 fraction and about 60% in the cerebellar P2 fraction, while the yield of NR1 was about 75% in the cortical P2 fraction and only about 40% in the cerebellar P2 fraction. The yields of both cytoskeletal proteins were 10% or less in both the cerebellar and cortical P2 fractions. Together these studies show that the P2 fractions contain glial and neuronal membrane proteins consistent with the earlier studies (25, 26).

**Table 1:** **Analyses of neuronal and astroglial proteins in tissue homogenate (TH) and various subcellular fractionations as described in Section “Materials and Methods**.”

	TH		S1		S2		P2	
	Enrichment	Yield	Enrichment	Yield	Enrichment	Yield	Enrichment	Yield
**Cortex**
GLT-1	1.0	100	1.1 ± 0.2	93 ± 20	0.44 ± 0.06	17 ± 2	1.2 ± 0.4	39 ± 13
GLAST	1.0	100	1.4 ± 0.3	112 ± 23	0.41 ± 0.07	15 ± 3	1.2 ± 0.4	39 ± 12
NR1	1.0	100	1.3 ± 0.2	107 ± 20	0.14 ± 0.005	5.2 ± 0.1	2.3 ± 0.4	74 ± 14
GFAP	1.0	100	0.41 ± 0.08	34 ± 7	0.5 ± 0.1	19 ± 4	0.26 ± 0.07	8 ± 2
NF-L	1.0	100	0.42 ± 0.09	34 ± 7	0.23 ± 0.04	9 ± 1	0.3 ± 0.1	10 ± 3
**Cerebellum**
GLT-1	1.0	100	1.3 ± 0.09	100 ± 5	0.4 ± 0.3	13 ± 8	2.7 ± 0.7	67 ± 20
GLAST	1.0	100	1.4 ± 0.05	108 ± 2	0.7 ± 0.1	22 ± 4	2.4 ± 0.6	59 ± 17
NR1	1.0	100	0.5 ± 0.2	36 ± 14	0.4 ± 0.3	14 ± 10	1.2 ± 0.1	37 ± 9
GFAP	1.0	100	0.37 ± 0.05	28 ± 4	0.57 ± 0.08	19 ± 2	0.18 ± 0.02	4.4 ± 0.7
NF-L	1.0	100	0.36 ± 0.08	27 ± 7	0.7 ± 0.2	24 ± 6	0.09 ± 0.02	2.1 ± 0.3

Enrichment and yield are normalized to the levels of the tissue homogenate as described in Section “Materials and Methods.” Data are the mean ± SEM of at least three independent measurements.

**Figure 2:**
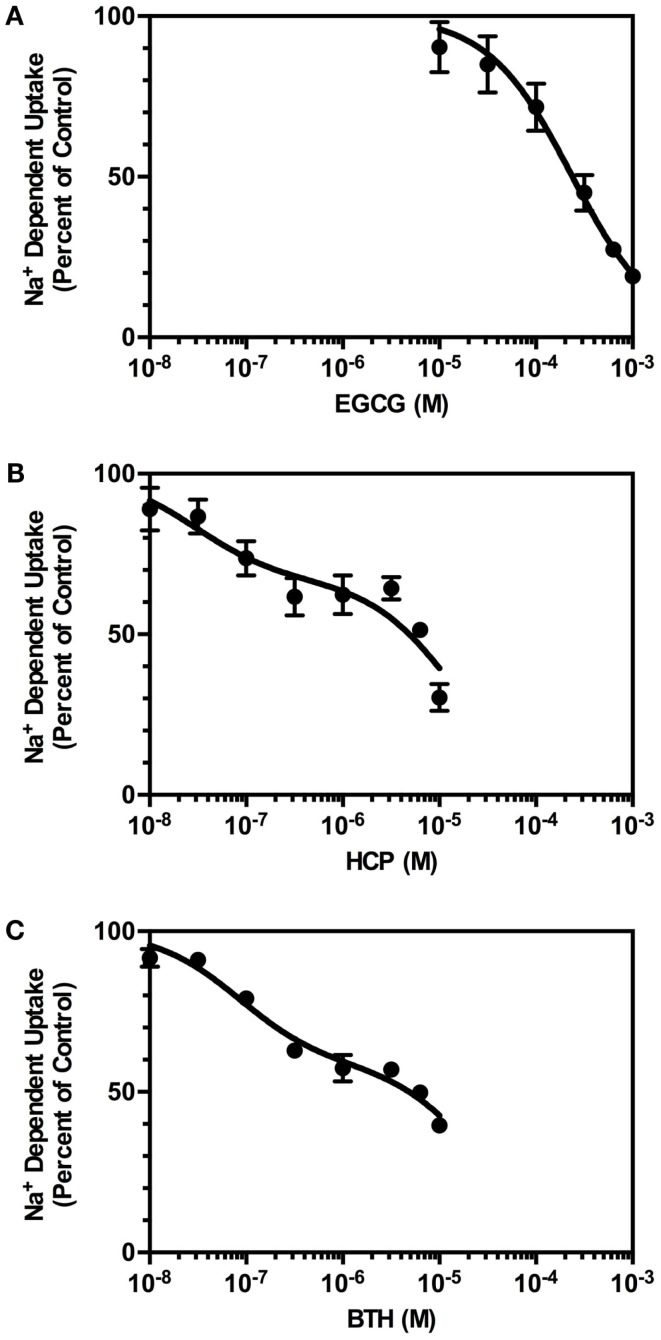
**Concentration-dependence of the effects of GDH inhibitors on of Na^+^-dependent l-[^3^H]-glutamate transport into crude membranes (P2) prepared from cortex**. Transport of l-[^3^H]-glutamate (0.5 μM) was measured in the absence or presence of increasing concentrations of inhibitors, as described in Section “Materials and Methods.” The percent of control represents the velocity of transport measured in the presence of inhibitor divided by that observed in the absence of inhibitor multiplied by 100. **(A)** Inhibition by EGCG was fit to a one-site competition model with an IC_50_ value of 234 μM. **(B)** Inhibition by HCP was fit to a two-site competition model. The IC_50_ value for the high affinity component was 30 nM, and the IC_50_ value for the lower affinity component was 14 μM. Based on this fit, 33% of the sites were high affinity and 67% were of lower affinity. **(C)** Inhibition by BTH was fit to a two-site competition model with an IC_50_ for fraction 1 (42%) of 84 nM and an IC_50_ for fraction 2 (58%) of 26 μM. Data are the mean ± SEM of at least three independent measurements (except for incubation of 1 μM BTH, of which there were only two observations).

In the first set of experiments, the effects of increasing concentrations of EGCG on Na^+^-dependent uptake were examined (Figure 2A). The effects of EGCG were concentration-dependent and inhibited uptake with an IC_50_ value of 234 μM. The maximal inhibition observed was 83% at 1 mM; higher concentrations were not tested because of solubility concerns. EGCG inhibits purified GDH with an IC_50_ value of ∼0.5 μM (21, 28), but EGCG is relatively hydrophilic and has limited stability in solution (29).

Several other inhibitors of GDH were recently identified in a high-throughput screen (28). We chose two additional inhibitors of GDH to reduce the likelihood that effects of EGCG on glutamate uptake might be attributed to a non-specific interaction with a target other than GDH. All three of these compounds interact with different sites on GDH (30). The first compound examined, hexachlorophene (HCP), caused a concentration-dependent inhibition of Na^+^-dependent glutamate uptake (Figure 2B). When these data were fit to a single site, the IC_50_ value was 3.9 μM, but the inhibition data were best fit to two sites with IC_50_ values of 30 nM and 14 μM. The maximal inhibition observed was 70% at 10 μM, and higher concentrations were not tested to avoid effects of solvent on uptake. The reported IC_50_ value for inhibition of GDH is 1.7 μM (28). We also examined the effects of bithionol (BTH), which caused a concentration-dependent inhibition of Na^+^-dependent glutamate transport activity in crude cortical membranes (P2; Figure 2C). The IC_50_ value was 4.1 μM when the data were fit to a single site, but the inhibition data were best fit to two sites with IC_50_ values of 84 nM and 26 μM. The maximal inhibition observed was 60% at 10 μM, and higher concentrations were not tested to avoid effects of solvent on uptake. The reported IC_50_ value for inhibition of GDH is 5.5 μM (28). Together, these studies show that three different inhibitors of GDH also inhibit Na^+^-dependent glutamate uptake in crude cortical membranes (P2).

**Figure 3:**
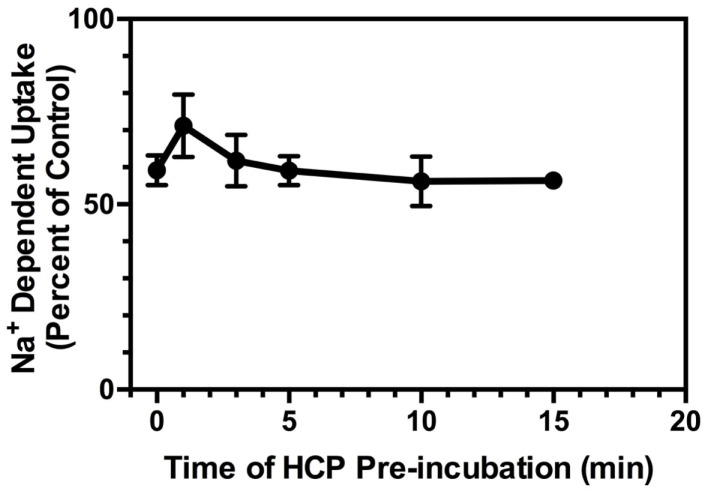
**Effects of pre-incubation with HCP (6 μM) on Na^+^-dependent l-[^3^H]-glutamate transport into crude membranes (P2) prepared from cortex**. HCP (or vehicle) was added to crude membranes (P2) at 37°C at 1, 3, 5, 10, or 15 min prior to the initiation of transport with the addition of l-[^3^H]-glutamate, or simultaneously with l-[^3^H]-glutamate (time = 0 min). Percent of control represents the velocity of transport in the presence of inhibitor divided by that observed in the absence of inhibitor multiplied by 100. Data are the mean ± SEM of at least three independent measurements.

One might expect that the effects of inhibition of GDH would increase with pre-incubation. To address this possibility, crude cortical membranes (P2) were pre-incubated with 6.0 μM HCP for up to 15 min prior to initiation of uptake by the addition of l-[^3^H]Glu. Somewhat surprisingly, the amount of inhibition observed with pre-incubations of 1, 3, 5, 10, and 15 min was not significantly different than that observed with no pre-incubation (Figure 3). In these experiments, the crude membranes (P2) were warmed to 37°C for the same amount of time, regardless of the length of the pre-incubation with HCP, to ensure the effects seen were independent of the increased time that the crude membranes (P2) were warmed to 37°C. This shows that the effects of HCP on glutamate uptake are very rapid and essentially instantaneous in this experimental paradigm where uptake is measured for 3 min.

**Figure 4:**
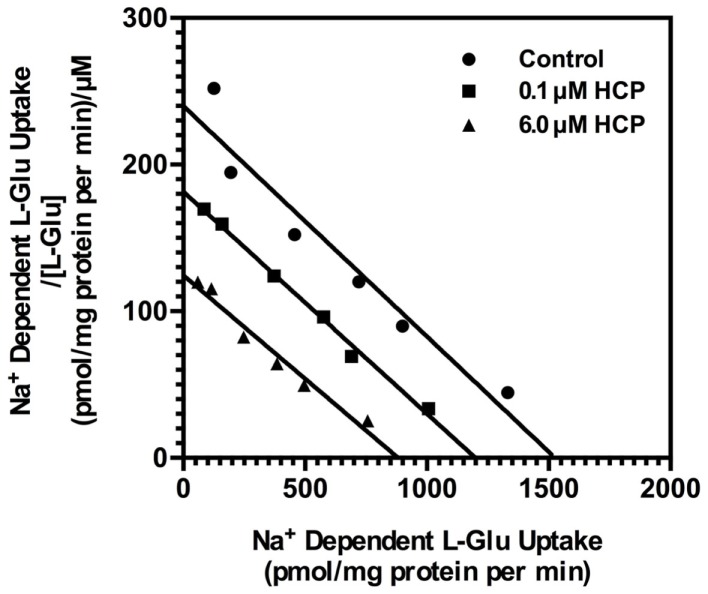
**Eadie–Hofstee plot of the concentration-dependence of l-[^3^H]-glutamate transport in crude membranes (P2) prepared from cortex with no treatment (control; circle), 0.1 μM HCP (square), or 6.0 μM HCP (triangle)**. Uptake was determined as described in Section “Materials and Methods.” Data presented are from a single experiment that has been repeated in four independent experiments with similar results. The *K*_m_ values for transport were 5.7 ± 0.3 μM in vehicle control, 5.4 ± 0.4 μM in the presence of 0.1 μM HCP, and 4.2 ± 1.0 μM in the presence of 6.0 μM HCP. The *V*_max_ values for transport were 1.6 ± 0.4 (nmol/mg protein/min) for vehicle control, 1.2 ± 0.4 in the presence of 0.1 μM HCP, and 0.7 ± 0.1 in the presence of 6.0 μM HCP. The *K*_m_ or *V*_max_ values were normalized to vehicle controls for each experiment (set to 100%) and compared to those observed in the presence of HCP. HCP had no significant effect on *K*_m_ value, but significantly reduced the *V*_max_ value at either 0.1 μM (*p* < 0.01) or 6 μM (*p* < 0.001).

To further characterize the mechanism of action, we examined the effects of HCP on the concentration-dependence for l-[^3^H]-Glu uptake. As was previously observed by us and others [for review, see (31)], the *K*_m_ for l-[^3^H]-Glu uptake was ∼5 μM and the *V*_max_ was ∼1.2 nmol/mg/min. HCP had no effect on the *K*_m_ value and decreased the *V*_max_ for l-[^3^H]-glutamate uptake (Figure 4). These data are consistent with a non-competitive mechanism of inhibition of glutamate uptake.

**Figure 5:**
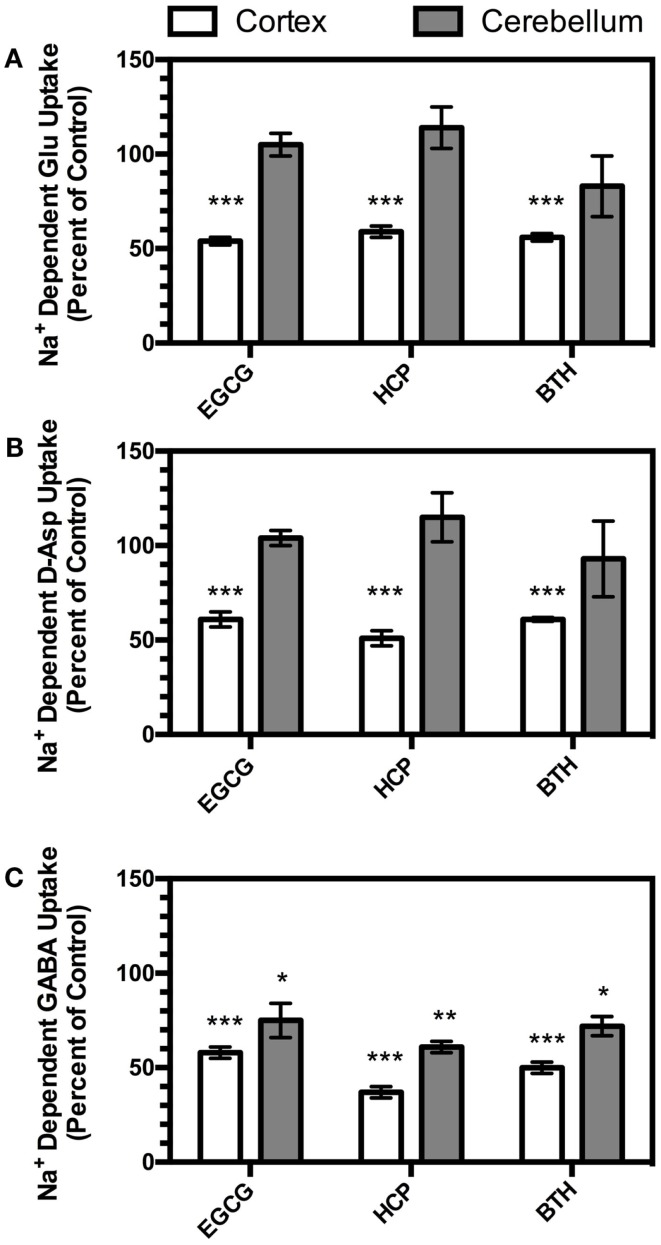
**Effects of EGCG (300 μM), HCP (6 μM), or BTH (3 μM) on Na^+^-dependent transport of L-glutamate (A), D-aspartate (B), or GABA (C) measured in crude membranes (P2) prepared from cortex (open bars) or cerebellum (shaded bars)**. Transport of all three substrates in both brain regions was measured in parallel experiments with different vehicle controls as described in the Section “Materials and Methods.” Data are the mean ± SEM from at least three independent experiments. Data were compared to vehicle controls by ANOVA. *Indicates a *p* < 0.05, ** indicates a *p* < 0.01, and *** indicates a *p* < 0.001.

Na^+^-dependent glutamate uptake into crude cerebellar membranes (P2) displays a dramatically different pharmacology from that observed in crude cortical membranes [P2; (20, 32)]. In fact, the pharmacology of transport in crude cerebellar membranes (P2) is consistent with that of GLAST (24). To determine if the effects of inhibition of GDH are selective for these two different transport activities, we compared the effects of HCP, EGCG, and BTH on Na^+^-dependent glutamate uptake in crude membranes (P2) prepared from cortex and cerebellum. None of the GDH inhibitors had any effect on Na^+^-dependent uptake in crude membranes (P2) prepared from cerebellum (Figure 5A).

d-Aspartate is transported by the Na^+^-dependent glutamate transporters (5), but is not a substrate for GDH (33). To determine if the effects of the GDH inhibitors might be related to direct metabolism of the glutamate that moves through the transporter during uptake, we examined the effects of the three different GDH inhibitors on d-[^3^H]-Asp uptake in crude membranes (P2) prepared from cortex and cerebellum. As was observed with l-[^3^H]-Glu transport, all three compounds inhibited d-[^3^H]-Asp uptake in crude cortical membranes (P2), and no effects were observed in cerebellar membranes (P2; Figure 5B).

The inhibitory neurotransmitter, γ-aminobutyric acid (GABA), is also cleared by Na^+^-dependent transport systems. The role of GDH in GABA metabolism is unclear, but it may play a role in the deamination of glutamate formed from the transamination of GABA by GABA-transaminase, also found in mitochondria [for discussions, see (34, 35)]. Therefore, the effects of the three different GDH inhibitors on Na^+^-dependent [^3^H]-GABA transport were examined in crude membranes (P2) prepared from cortex and cerebellum. As was observed with l-[^3^H]-Glu or d-[^3^H]-Asp, all three GDH inhibitors reduced Na^+^-dependent [^3^H]-GABA transport in cortical membranes (P2; Figure 5C). However, in contrast to the lack of inhibition observed with l-[^3^H]-Glu or d-[^3^H]-Asp, all three compounds also inhibited [^3^H]-GABA transport in crude cerebellar membranes (P2).

## Discussion

In a recent study, we obtained preliminary evidence that inhibition of GDH might lead to inhibition of Na^+^-dependent glutamate uptake (19). In the present study, we observed concentration-dependent inhibition of Na^+^-dependent glutamate uptake in crude membranes (P2) prepared from cortical tissue using three different inhibitors of GDH. The effects of one of these inhibitors, HCP, were studied further. As pre-incubation did not increase the effects of HCP, inhibition occurs relatively rapidly (within seconds to minutes). HCP had no effect on *K*_m_ for glutamate uptake, but reduced *V*_max_, consistent with a non-competitive mechanism of action. All three compounds also inhibited Na^+^-dependent d-[^3^H]-Asp and [^3^H]-GABA to a similar extent in crude cortical membranes (P2). However, in crude membranes (P2) prepared from cerebellum, these compounds did not affect Na^+^-dependent l-[^3^H]-Glu or d-[^3^H]-Asp, but did inhibit [^3^H]-GABA transport.

The fact that three different inhibitors of GDH also block Na^+^-dependent glutamate and GABA uptake suggests that the observed inhibition is likely to be due to an effect on GDH. The potencies of HCP or BTH for inhibition of glutamate uptake are similar to those observed for inhibition of GDH (all in the micromolar range – see Results). One concern is that the concentration of EGCG required to inhibit transport is approximately 500-fold higher than that required to inhibit GDH; one major difference is that the analyses of GDH were conducted with purified enzyme (21). Others have shown that the EGCG is not particularly stable in solution, is subject to active efflux from some cellular systems by ABC cassette proteins, and may be poorly absorbed across membranes (29, 36). Therefore, it is possible that this difference in potency simply reflects the fact that glutamate uptake was measured in an intact system. Based on analyses of crystal structures of GDH, HCP and BTH bind to distinct locations in the interior of the GDH hexamer (30). Based on analyses with epicatechin 3-monogallate (an EGCG analog), it seems that EGCG likely binds to the ADP-activation site, located by the pivot helix of GDH (37). We also considered the possibility that these compounds might block glutamate uptake through a direct interaction with GLT-1, but do not think this is likely for two different reasons. First, the fact that three different inhibitors have the same effect on uptake makes this a less likely explanation. Second, all three inhibitors block sodium-dependent GABA uptake as well, which is mediated by a completely distinct family of transporters that share no homology with the glutamate transporters [for review, see (38)]. Therefore, we suggest that the simplest explanation for the present results is that inhibition of GDH rapidly inhibits Na^+^-dependent glutamate or GABA uptake.

In the present study, we found that inhibition of GDH had no effect on l-glutamate or d-aspartate transport in crude cerebellar membranes (P2) where GLAST likely mediates uptake (20, 24, 32). Yet, we previously found that EGCG inhibits glutamate uptake in astrocyte cultures (19) where uptake is also mediated by GLAST (22, 23, 39). These measures of transport were conducted in identical buffers; this suggests that the differential effects cannot be attributed to the utilization of different exogenous metabolites to generate ATP. Coupling of GDH to transport may vary depending on the maturation of the system; astrocytes in culture are polygonal, not stellate shaped as is observed *in vivo*, and do not express GLT-1 consistent with an immature phenotype (22, 23). While maturation may explain the difference between astrocytes in culture and crude cerebellar membranes (P2), GDH has been recently deleted from the CNS tissues in mice (40) and also been knocked down in astrocytes in culture (41). Interestingly, the mice with the CNS specific deletion of GDH display no overt behavioral phenotype, and one would predict a seizure phenotype if deletion of GDH resulted in impaired glutamate uptake *in vivo* (42, 43). Therefore, as has been observed with differential metabolism of glutamate in different systems or with increased neuronal activity [for reviews, see (44, 45)], it seems likely that redundant systems may be differentially engaged to support glutamate uptake.

In the present study, we used crude membranes (P2). Previous studies have demonstrated that the pharmacology of glutamate uptake in cortical crude membranes parallels that observed for GLT-1 and is distinct from that observed in cerebellar crude membranes uptake (20, 24, 32). Furthermore, genetic deletion of GLT-1 reduces glutamate uptake in cortical crude membranes to 5% of control (9). These studies provide compelling evidence that uptake measured in cortical crude membranes is mediated by GLT-1 and that uptake measured in cerebellar crude membranes is mediated by GLAST. We examined the abundance of a neuronal membrane protein, the NR1 subunit of the NMDA receptor, and two glial membrane proteins, GLT-1 and GLAST in this membrane preparation. As was previously observed (25, 26), we found that this fraction contains high levels of neuronal and glial membrane proteins. We also found low levels of the cytoskeletal proteins, GFAP and NF-L. The absence of large amounts of either cytoskeletal protein suggests that the membranes do not uniformly reseal into vesicles and that the resealed vesicles contain only a small fraction of the original cytosol. This conclusion is also supported by the observation that cortical and cerebellar crude membranes contain comparable levels of GLT-1 (Figure 1), even though no GLT-1-mediated uptake is detected in cerebellar crude membranes (see above).

GLT-1 expression, when analyzed using immunohistochemical approaches (7, 15, 16), *in situ* hybridization of mRNA [(46, 47), and for reviews, see (4, 5)], or bacterial artificial chromosome GLT-1 promoter reporter mice (48), is essentially restricted to astrocytes. However, there is also evidence that GLT-1 is expressed in some neurons in the CNS (8, 47, 49). The P2 membrane preparation has also been used to examine the relative contributions of the different GABA transporters to uptake. The pharmacology of GABA transport in crude membranes (P2) is consistent with GAT-1 (50, 51), which is thought to be localized predominantly in neurons, but is also found in astrocytes [for review, see (52)]. Although GLT-1 is heavily enriched in astrocytes and GAT-1 is enriched in neurons, it is not possible to determine if the effects of inhibition of GDH are related to inhibition of neuronal or glial pools of this enzyme. This will need to be a topic of further investigation.

Glutamate dehydrogenase is a mitochondrial enzyme that catalyzes the reversible deamination of glutamate to α-ketoglutarate [for reviews, see (53, 54)]. This reaction is accompanied by the production of NADH that can be used to generate ATP; the downstream metabolism of α-ketoglutarate through the tricarboxylic acid cycle will also generate ATP. Cytoplasmic glutamate moves into mitochondria by one of two different families of transporters; neither of these processes transport d-aspartate (55, 56). Furthermore, GDH does not metabolize d-Asp (33). Therefore, although it is theoretically possible that GDH might support inward transport by rapidly metabolizing glutamate and thereby maintaining a concentration gradient, this is unlikely because inhibition of GDH also blocks transport of d-[^3^H]-Asp. Others have found that synaptosomes enriched from P2 contain relatively high levels of endogenous glutamate (57); therefore, it seems certain that the crude membranes (P2) used in the present study contain endogenous glutamate. Combined with the fact that these inhibitors also block GABA transport, these observations suggest that mitochondrial GDH provides energy for transport using endogenous glutamate to drive the Na^+^-dependent transport systems.

Several studies have demonstrated that inhibition of mitochondrial function impairs glutamate uptake. For example, the mitochondrial poisons, sodium azide, dinitrophenol, and antimycin A, inhibit glutamate uptake in cultured astrocytes (58). In addition, MPP^+^ and rotenone, inhibitors of complex 1 of the electron transport chain, inhibit glutamate clearance in both astrocytes and crude cerebral synaptosomes (59, 60). Opening mitochondrial K_ATP_ channels can functionally support glutamate uptake in the presence of MPP^+^ (61). There is also evidence that glutamate transport couples to ionic changes in mitochondria. In fact, uptake is accompanied by an increase in mitochondrial Na^+^ (62). Uptake is also accompanied by acidification of mitochondria (63). This may be dependent upon the mitochondrial glutamate–aspartate exchanger, Aralar, which co-transports a H^+^ with glutamate (55), or the mitochondrial glutamate carrier, which transports glutamate with a H^+^ or in exchange for a hydroxyl ion (56). The fact that inhibitors of GDH block glutamate uptake provides further support for a functional interaction between transporters and mitochondria.

Epigallocatechin-monogallate is considered the most active of the green tea polyphenols, the likely active ingredients in green tea; the effects of EGCG on biological/pathological processes have been examined in numerous *in vitro* and *in vivo* studies. EGCG is of particular interest in the treatment of Alzheimer’s and Parkinson’s diseases because it exhibits neuroprotective effects such as radical scavenging, iron chelating, activation of PKC, and anti-apoptotic action [for review, see (64)]. *In vivo* studies have demonstrated the neuroprotective properties of EGCG. For example, these polyphenols improve spatial learning in aged rodents (65), they suppress peripheral nerve degeneration associated with sciatic nerve crush (66), and EGCG protects against hippocampal neuronal damage after global ischemia (67). *In vitro*, EGCG is protective at low concentrations, but at concentrations similar to those used in the present study, EGCG causes cell death in a neuroblastoma cell line (68). EGCG increases the amount of glutamate observed in the effluent upon depolarization of synaptosomes (69). In pancreatic β-cells, EGCG inhibits the increase in insulin secretion and glutamine oxidation caused by the stimulation of GDH (21). In most of these examples, it is unclear if the observed effects are related to inhibition of GDH or to the anti-oxidant activity of EGCG. Inhibition of GDH and glutamate uptake might contribute to some of the observed *in vivo* effects, but this will need to be resolved in future studies.

In summary, we show evidence that inhibition of the mitochondrial enzyme, GDH, can result in inhibition of Na^+^-dependent l-glutamate or d-aspartate uptake in mammalian cortex. It seems likely that this dependence on GDH is affected by factors that are yet to be defined.

## Conflict of Interest Statement

The authors declare that the research was conducted in the absence of any commercial or financial relationships that could be construed as a potential conflict of interest.

## References

[1] Sheng M Hoogenraad CC The postsynaptic architecture of excitatory synapses: a more quantitative view. Annu Rev Biochem (2007) 76:823–47 10.1146/annurev.biochem.76.060805.160029 17243894

[2] Doble A The role of excitotoxicity in neurodegenerative disease: implications for therapy. Pharmacol Ther (1999) 81:163–221 10.1016/S0163-7258(98)00042-4 10334661

[3] Olney J Excitotoxicity, apoptosis and neuropsychiatric disorders. Curr Opin Pharmacol (2003) 3:101–9 10.1016/S1471489202000024 12550750

[4] Sims KD Robinson MB Expression patterns and regulation of glutamate transporters in the developing and adult nervous system. Crit Rev Neurobiol (1999) 13:169–97 10512489 10.1615/critrevneurobiol.v13.i2.30

[5] Danbolt NC Glutamate uptake. Prog Neurobiol (2001) 65:1–105 10.1016/S0301-0082(00)00067-8 11369436

[6] Sheldon AL Robinson MB The role of glutamate transporters in neurodegenerative diseases and potential opportunities for intervention. Neurochem Int (2007) 51:333–55 10.1016/j.neuint.2007.03.012 17517448 PMC2075474

[7] Rothstein JD Martin L Levey AI Dykes-Hoberg M Jin L Wu D Localization of neuronal and glial glutamate transporters. Neuron (1994) 13:713–25 10.1016/0896-6273(94)90038-8 7917301

[8] Chen W Mahadomrongkul V Berger UV Bassan M Desilva T Tanaka K The glutamate transporter GLT1a is expressed in excitatory terminals of mature hippocampal neurons. J Neurosci (2004) 24:1136–48 10.1523/JNEUROSCI.1586-03.2004 14762132 PMC2849838

[9] Tanaka K Watase K Manabe T Yamada K Watanabe M Takahashi K Epilepsy and exacerbation of brain injury in mice lacking the glutamate transporter GLT-1. Science (1997) 276:1699–702 10.1126/science.276.5319.1699 9180080

[10] Robinson MB The family of sodium-dependent glutamate transporters: a focus on the GLT-1/EAAT2 subtype. Neurochem Int (1999) 33:479–91 10.1016/S0197-0186(98)00055-2 10098717

[11] Torres GE Gainetdinov RR Caron MG Plasma membrane monoamine transporters: structure, regulation and function. Nat Rev Neurosci (2003) 4:13–25 10.1038/nrn1008 12511858

[12] Gether U Andersen PH Larsson OM Schousboe A Neurotransmitter transporters: molecular function of important drug targets. Trends Pharmacol Sci (2006) 27:375–83 10.1016/j.tips.2006.05.003 16762425

[13] Zerangue N Kavanaugh MP Flux coupling in a neuronal glutamate transporter. Nature (1996) 383:634–7 10.1038/383634a0 8857541

[14] Attwell D Gibb A Neuroenergetics and the kinetic design of excitatory synapses. Nat Rev Neurosci (2005) 6:841–9 10.1038/nrn1784 16261178

[15] Chaudhry FA Lehre KP Campagne MVL Ottersen OP Danbolt NC Storm-Mathisen J Glutamate transporters in glial plasma membranes: highly differentiated localizations revealed by quantitative ultrastructural immunocytochemistry. Neuron (1995) 15:711–20 10.1016/0896-6273(95)90158-2 7546749

[16] Lehre KP Levy LM Ottersen OP Storm-Mathisen J Danbolt NC Differential expression of two glial glutamate transporters in the rat brain: quantitative and immunocytochemical observations. J Neurosci (1995) 15:1835–53 7891138 10.1523/JNEUROSCI.15-03-01835.1995 PMC6578153

[17] Genda EN Jackson JG Sheldon AL Locke SF Greco TM O’Donnell JC Co-compartmentalization of the astroglial glutamate transporter, GLT-1, with glycolytic enzymes and mitochondria. J Neurosci (2011) 31:18275–88 10.1523/JNEUROSCI.3305-11.2011 22171032 PMC3259858

[18] Rose EM Koo JC Antflick JE Ahmed SM Angers S Hampson DR Glutamate transporter coupling to Na,K-ATPase. J Neurosci (2009) 29:8143–55 10.1523/JNEUROSCI.1081-09.2009 19553454 PMC6666056

[19] Bauer DE Jackson JG Genda EN Montoya MM Yudkoff M Robinson MB The glutamate transporter, GLAST, participates in a macromolecular complex that supports glutamate metabolism. Neurochem Int (2012) 61:566–74 22306776 10.1016/j.neuint.2012.01.013 PMC3350823

[20] Robinson MB Hunter-Ensor M Sinor J Pharmacologically distinct sodium-dependent L-[^3^H]glutamate transport processes in rat brain. Brain Res (1991) 544:196–202 10.1016/0006-8993(91)90054-Y 2039937

[21] Li C Allen A Kwagh J Doliba NM Qin W Najafi H Green tea polyphenols modulate insulin secretion by inhibiting glutamate dehydrogenase. J Biol Chem (2006) 281:10214–21 10.1074/jbc.M512792200 16476731

[22] Swanson RA Liu J Miller JW Rothstein JD Farrell K Stein BA Neuronal regulation of glutamate transporter subtype expression in astrocytes. J Neurosci (1997) 17:932–40 8994048 10.1523/JNEUROSCI.17-03-00932.1997 PMC6573161

[23] Schlag BD Vondrasek JR Munir M Kalandadze A Zelenaia OA Rothstein JD Regulation of the glial Na^+^-dependent glutamate transporters by cyclic AMP analogs and neurons. Mol Pharmacol (1998) 53:355–69 9495799 10.1124/mol.53.3.355

[24] Arriza JL Fairman WA Wadiche JI Murdoch GH Kavanaugh MP Amara SG Functional comparisons of three glutamate transporter subtypes cloned from human motor cortex. J Neurosci (1994) 14:5559–69 7521911 10.1523/JNEUROSCI.14-09-05559.1994 PMC6577102

[25] Henn FA Anderson DJ Rustad DG Glial contamination of synaptosomal fractions. Brain Res (1976) 101:341–4 10.1016/0006-8993(76)90274-2 1244976

[26] Gylys KH Fein JA Cole GM Quantitative characterization of crude synaptosomal fraction (P-2) components by flow cytometry. J Neurosci Res (2000) 61:186–92 10.1002/1097-4547(20000715)61:2<186::AID-JNR9>3.0.CO;2-X 10878591

[27] Fuchs E Weber K Intermediate filaments: structure, dynamics, function, and disease. Annu Rev Biochem (1994) 63:345–82 10.1146/annurev.bi.63.070194.002021 7979242

[28] Li M Allen A Smith TJ High throughput screening reveals several new classes of glutamate dehydrogenase inhibitors. Biochemistry (2007) 46:15089–102 10.1021/bi7018783 18044977 PMC2440342

[29] Hong J Lu H Meng X Ryu JH Hara Y Yang CS Stability, cellular uptake, biotransformation, and efflux of tea polyphenol (-)-epigallocatechin-3-gallate in HT-29 human colon adenocarcinoma cells. Cancer Res (2002) 62:7241–6 12499265

[30] Li M Smith CJ Walker MT Smith TJ Novel inhibitors complexed with glutamate dehydrogenase: allosteric regulation by control of protein dynamics. J Biol Chem (2009) 284:22988–3000 10.1074/jbc.M109.020222 19531491 PMC2755706

[31] Robinson MB Dowd LA Heterogeneity and functional properties of subtypes of sodium-dependent glutamate transporters in the mammalian central nervous system. Adv Pharmacol (1997) 37:69–115 10.1016/S1054-3589(08)60948-5 8891100

[32] Ferkany J Coyle JT Heterogeneity of sodium-dependent excitatory amino acid uptake mechanisms in rat brain. J Neurosci Res (1986) 16:491–503 10.1002/jnr.490160305 2877096

[33] Fosse VM Kolstad J Fonnum F A bioluminescence method for the measurement of L-glutamate: applications to the study of changes in the release of L-glutamate from lateral geniculate nucleus and superior colliculus after visual cortex ablation in rats. J Neurochem (1986) 47:340–9 10.1111/j.1471-4159.1986.tb04507.x 2874187

[34] Schousboe I Bro B Schousboe A Intramitochondrial localization of the 4-aminobutyrate-2-oxoglutarate transaminase from ox brain. Biochem J (1977) 162:303–7 849286 10.1042/bj1620303 PMC1164602

[35] Kreft M Bak LK Waagepetersen HS Schousboe A Aspects of astrocyte energy metabolism, amino acid neurotransmitter homoeostasis and metabolic compartmentation. ASN Neuro (2012) 4:187–99 10.1042/AN20120007 22435484 PMC3338196

[36] Zhong Y Chiou YS Pan MH Shahidi F Anti-inflammatory activity of lipophilic epigallocatechin gallate (EGCG) derivatives in LPS-stimulated murine macrophages. Food Chem (2012) 134:742–8 10.1016/j.foodchem.2012.02.172 23107686

[37] Li C Li M Chen P Narayan S Matschinsky FM Bennett MJ Green tea polyphenols control dysregulated glutamate dehydrogenase in transgenic mice by hijacking the ADP activation site. J Biol Chem (2011) 286:34164–74 10.1074/jbc.M111.268599 21813650 PMC3190766

[38] Nelson N The family of Na^+^/Cl^-^ neurotransmitter transporters. J Neurochem (1998) 71:1785–803 10.1046/j.1471-4159.1998.71051785.x 9798903

[39] Garlin AB Sinor AD Sinor JD Jee SH Grinspan JB Robinson MB Pharmacology of sodium-dependent high-affinity L-[^3^H]glutamate transport in glial cultures. J Neurochem (1995) 64:2572–80 10.1046/j.1471-4159.1995.64062572.x 7760037

[40] Frigerio F Karaca M De Roo M Mlynarik V Skytt DM Carobbio S Deletion of glutamate dehydrogenase 1 (Glud1) in the central nervous system affects glutamate handling without altering synaptic transmission. J Neurochem (2012) 123:342–8 10.1111/j.1471-4159.2012.07933.x 22924626

[41] Skytt DM Klawonn AM Stridh MH Pajecka K Patruss Y Quintana-Cabrera R siRNA knock down of glutamate dehydrogenase in astrocytes affects glutamate metabolism leading to extensive accumulation of the neuroactive amino acids glutamate and aspartate. Neurochem Int (2012) 61:490–7 10.1016/j.neuint.2012.04.014 22542772

[42] Demarque M Villeneuve N Manent JB Becq H Represa A Ben-Ari Y Glutamate transporters prevent the generation of seizures in the developing rat neocortex. J Neurosci (2004) 24:3289–94 10.1523/JNEUROSCI.5338-03.2004 15056708 PMC6730015

[43] Shimamoto K Sakai R Takaoka K Yumoto N Nakajima T Amara SG Characterization of novel L-threo-β-benzyloxy aspartate derivatives, potent blockers of the glutamate transporters. Mol Pharmacol (2004) 65:1008–15 10.1124/mol.65.4.1008 15044631

[44] Schousboe A Westergaard N Sonnewald U Petersen SB Huang R Peng L Glutamate and glutamine metabolism and compartmentation in astrocytes. Dev Neurosci (1993) 15:359–66 10.1159/000111356 7805590

[45] McKenna MC The glutamate-glutamine cycle is not stoichiometric: fates of glutamate in brain. J Neurosci Res (2007) 85:3347–58 10.1002/jnr.21444 17847118

[46] Torp R Danbolt NC Babaie E Bjoras M Seeberg E Storm-Mathisen J Differential expression of two glial glutamate transporters in the rat brain: an *in situ* hybridization study. Eur J Neurosci (1994) 6:936–42 10.1111/j.1460-9568.1994.tb00587.x 7952280

[47] Schmitt A Asan E Puschel B Jons T Kugler P Expression of the glutamate transporter GLT1 in neural cells of the rat central nervous system: non-radioactive *in situ* hybridization and comparative immunocytochemistry. Neuroscience (1996) 71:989–1004 10.1016/0306-4522(95)00477-7 8684627

[48] Regan MR Huang YH Kim YS Dykes-Hoberg MI Jin L Watkins AM Variations in promoter activity reveal a differential expression and physiology of glutamate transporters by glia in the developing and mature CNS. J Neurosci (2007) 27:6607–19 10.1523/JNEUROSCI.0790-07.2007 17581948 PMC6672708

[49] Furness DN Dehnes Y Akhtar AQ Rossi DJ Hamann M Grutle NJ A quantitative assessment of glutamate uptake into hippocampal synaptic terminals and astrocytes: new insights into a neuronal role for excitatory amino acid transporter 2 (EAAT2). Neuroscience (2008) 157:80–94 10.1016/j.neuroscience.2008.08.043 18805467 PMC2775085

[50] Sutch RJ Davies CC Bowery NG GABA release and uptake measured in crude synaptosomes from genetic absence epilepsy rats from Strasbourg (GAERS). Neurochem Int (1999) 34:415–25 10.1016/S0197-0186(99)00046-7 10397370

[51] Ghasemi A Sadidi A Mohammadi M Khoshbaten A Asgari A Paraoxon inhibits GABA uptake in brain synaptosomes. Toxicol In vitro (2007) 21:1499–504 10.1016/j.tiv.2007.06.009 17686608

[52] Borden LA GABA transporter heterogeneity: pharmacology and cellular localization. Neurochem Int (1996) 29:335–56 10.1016/0197-0186(95)00158-1 8939442

[53] Plaitakis A Zaganas I Regulation of human glutamate dehydrogenases: implications for glutamate, ammonia and energy metabolism in brain. J Neurosci Res (2001) 66:899–908 10.1002/jnr.10054 11746417

[54] Li M Li C Allen A Stanley CA Smith TJ The structure and allosteric regulation of mammalian ]. Arch Biochem Biophys (2012) 519:69–80 10.1016/j.abb.2011.10.015 22079166 PMC3294041

[55] Palmieri L Pardo B Lasorsa FM Del Arco A Kobayashi K Iijima M Citrin and aralar1 are Ca(2+)-stimulated aspartate/glutamate transporters in mitochondria. EMBO J (2001) 20:5060–9 10.1093/emboj/20.18.5060 11566871 PMC125626

[56] Fiermonte G Palmieri L Todisco S Agrimi G Palmieri F Walker JE Identification of the mitochondrial glutamate transporter. Bacterial expression, reconstitution, functional characterization, and tissue distribution of two human isoforms. J Biol Chem (2002) 277:19289–94 10.1074/jbc.M201572200 11897791

[57] Erecinska M Zaleska MM Nissim I Nelson D Dagani F Yudkoff M Glucose and synaptosomal glutamate metabolism: studies with [15N]glutamate. J Neurochem (1988) 51:892–902 10.1111/j.1471-4159.1988.tb01826.x 2900879

[58] Swanson RA Farrell K Simon RP Acidosis causes failure of astrocyte glutamate uptake during hypoxia. J Cereb Blood Flow Metab (1995) 15:417–24 10.1038/jcbfm.1995.52 7713999

[59] Di Monte DA Tokar I Langston JW Impaired glutamate clearance as a consequence of energy failure caused by MPP(+) in astrocytic cultures. Toxicol Appl Pharmacol (1999) 158:296–302 10.1006/taap.1999.8717 10438663

[60] Yang YL Meng CH Ding JH He HR Ellsworth K Wu J Iptakalim hydrochloride protects cells against neurotoxin-induced glutamate transporter dysfunction in in vitro and in vivo models. Brain Res (2005) 1049:80–8 10.1016/j.brainres.2005.04.073 15932749

[61] Sun XL Zeng XN Zhou F Dai CP Ding JH Hu G KATP channel openers facilitate glutamate uptake by GluTs in rat primary cultured astrocytes. Neuropsychopharmacology (2008) 33:1336–42 10.1038/sj.npp.1301501 17609675

[62] Bernardinelli Y Azarias G Chatton JY In situ fluorescence imaging of glutamate-evoked mitochondrial Na+ responses in astrocytes. Glia (2006) 54:460–70 10.1002/glia.20387 16886210

[63] Azarias G Perreten H Lengacher S Poburko D Demaurex N Magistretti PJ Glutamate transport decreases mitochondrial pH and modulates oxidative metabolism in astrocytes. J Neurosci (2011) 31:3550–9 10.1523/JNEUROSCI.4378-10.2011 21389211 PMC6622778

[64] Mandel S Youdim MB Catechin polyphenols: neurodegeneration and neuroprotection in neurodegenerative diseases. Free Radic Biol Med (2004) 37:304–17 10.1016/j.freeradbiomed.2004.04.012 15223064

[65] Haque AM Hashimoto M Katakura M Tanabe Y Hara Y Shido O Long-term administration of green tea catechins improves spatial cognition learning ability in rats. J Nutr (2006) 136:1043–7 16549472 10.1093/jn/136.4.1043

[66] Renno WM Al-Maghrebi M Alshammari A George P (-)-Epigallocatechin-3-gallate (EGCG) attenuates peripheral nerve degeneration in rat sciatic nerve crush injury. Neurochem Int (2013) 62:221–31 10.1016/j.neuint.2012.12.018 23313191

[67] Lee S Suh S Kim S Protective effects of the green tea polyphenol (-)-epigallocatechin gallate against hippocampal neuronal damage after transient global ischemia in gerbils. Neurosci Lett (2000) 287:191–4 10.1016/S0304-3940(00)01159-9 10863027

[68] Levites Y Amit T Youdim MB Mandel S Involvement of protein kinase C activation and cell survival/cell cycle genes in green tea polyphenol (-)-epigallocatechin 3-gallate neuroprotective action. J Biol Chem (2002) 277:30574–80 10.1074/jbc.M202832200 12058035

[69] Chou CW Huang WJ Tien LT Wang SJ (-)-Epigallocatechin gallate, the most active polyphenolic catechin in green tea, presynaptically facilitates Ca2+-dependent glutamate release via activation of protein kinase C in rat cerebral cortex. Synapse (2007) 61:889–902 10.1002/syn.20444 17663453

